# Real-time cardiovascular magnetic resonance imaging for non-invasive characterisation of heart failure with preserved ejection fraction: final outcomes of the HFpEF stress trial

**DOI:** 10.1007/s00392-023-02363-5

**Published:** 2024-01-03

**Authors:** Sören J. Backhaus, Alexander Schulz, Torben Lange, Lennart S. Schmidt-Schweda, Ruben Evertz, Johannes Kowallick, Gerd Hasenfuß, Andreas Schuster

**Affiliations:** 1 Department of Cardiology and Pneumology, University Medical Center Göttingen, Georg-August-University Göttingen, Robert-Koch-Str. 40, 37099 Göttingen, Germany; 2 German Center for Cardiovascular Research (DZHK), Partner Site Göttingen, Göttingen, Germany; 3 Department of Diagnostic and Interventional Radiology, University Medical Center Göttingen, Göttingen, Germany

**Keywords:** HFpEF, Real-time cardiovascular magnetic resonance, Exercise-stress, Atrial function, Deformation, Strain

## Abstract

**Background:**

The diagnosis of heart failure with preserved ejection fraction (HFpEF) remains challenging. Recently, the HFpEF Stress Trial demonstrated feasibility and accuracy of non-invasive cardiovascular magnetic resonance (CMR) real-time (RT) exercise-stress atrial function imaging for early identification of HFpEF. However, no outcome data have yet been presented.

**Methods:**

The HFpEF Stress Trial (DZHK-17) prospectively recruited 75 patients with dyspnea on exertion and echocardiographic preserved EF and signs of diastolic dysfunction (*E*/*e*ʹ > 8). 68 patients entered the final study cohort and were characterized as HFpEF (*n = *34) or non-cardiac dyspnea (*n = *34) according to pulmonary capillary wedge pressure (HFpEF: PCWP rest: ≥ 15 mmHg stress: ≥ 25 mmHg). These patients were contacted by telephone and hospital charts were reviewed. The clinical endpoint was cardiovascular events (CVE).

**Results:**

Follow-up was performed after 48 months; 1 patient was lost to follow-up. HFpEF patients were more frequently compared to non-cardiac dyspnea (15 vs. 8, *p = *0.059). Hospitalised patients during follow-up had higher H2FPEF scores (5 vs. 3, *p < *0.001), and impaired left atrial (LA) function at rest (*p ≤ *0.002) and stress (*p ≤ *0.006). Impairment of CMR-derived atrial function parameters at rest and during exercise-stress (*p ≤ *0.003) was associated with increased likelihood for CVE. CMR-Feature Tracking LA Es/Ee (*p = *0.016/0.017) and RT-CMR derived LA long axis strain (*p = *0.003) were predictors of CVE independent of the presence of atrial fibrillation.

**Conclusions:**

Left atrial function emerged as the strongest predictor for 4-year outcome in the HFpEF Stress Trial. A combination of rest and exercise-stress LA function quantification allows accurate diagnostic and prognostic stratification in HFpEF.

Clinicaltrials.gov: NCT03260621.

**Graphical abstract:**

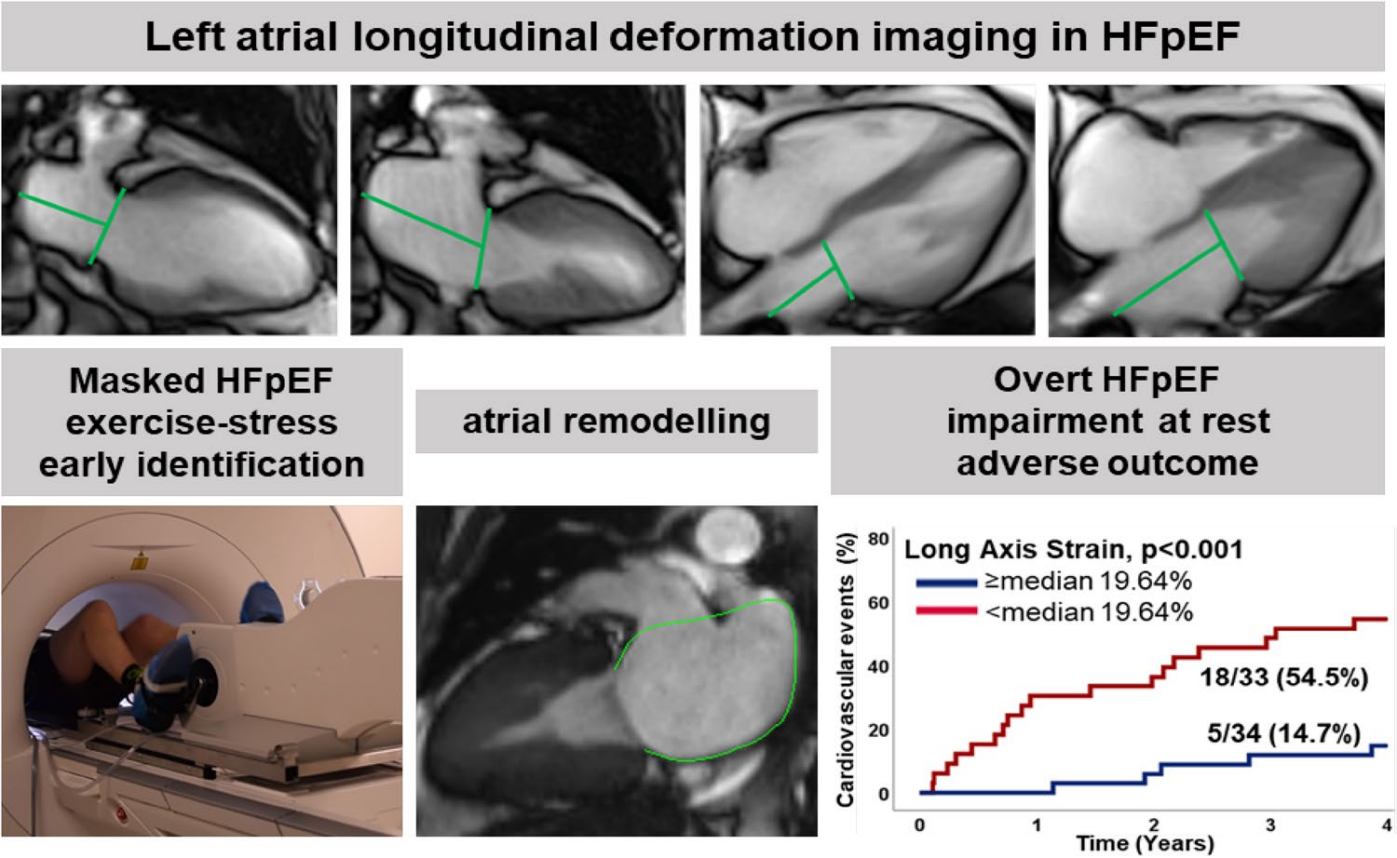

## Introduction

Heart failure with preserved or mildly reduced ejection fraction (HFpEF) accounts for more than half of the heart failure population [1]. The heterogenous pathophysiology and late symptom onset not only delay the diagnosis of HFpEF [2] but complicate targeted therapeutic decisions [3–5]. For early identification of diastolic dysfunction and decision making in uncertain cases, current guidelines recommend invasive right heart catheterization (RHC) including exercise-stress testing [6, 7]. Especially the identification of an early disease stage may critically advance efforts in the prevention or delay of cardiac remodelling and clinical deterioration from diastolic dysfunction [4, 5, 8–10]. The invasive nature of RHC as well as challenging examination conditions especially using echocardiography [11] during physiological exercise-stress have been impediments to the widespread adoption of exercise-stress protocols to the clinical routine.

Recently, the HFpEF Stress Trial [12] has demonstrated high diagnostic accuracy of cardiovascular magnetic resonance imaging (CMR) real-time (RT) exercise-stress testing at high temporal resolution for the non-invasive diagnosis of HFpEF [13]. While this diagnostic methodology has shown promise for the diagnosis of HFpEF, data on its prognostic implications are scarce. The medium-term follow-up of the HFpEF Stress trial [14] demonstrated the impact of atrial strain for prognostic assessment. Given slow disease progress in HFpEF and initial early diagnosis due to exercise-stress testing, the present long-term follow-up aimed for higher numbers in cardiovascular events (CVE) for improved statistical evaluation.

## Methods

**Fig. 1:**
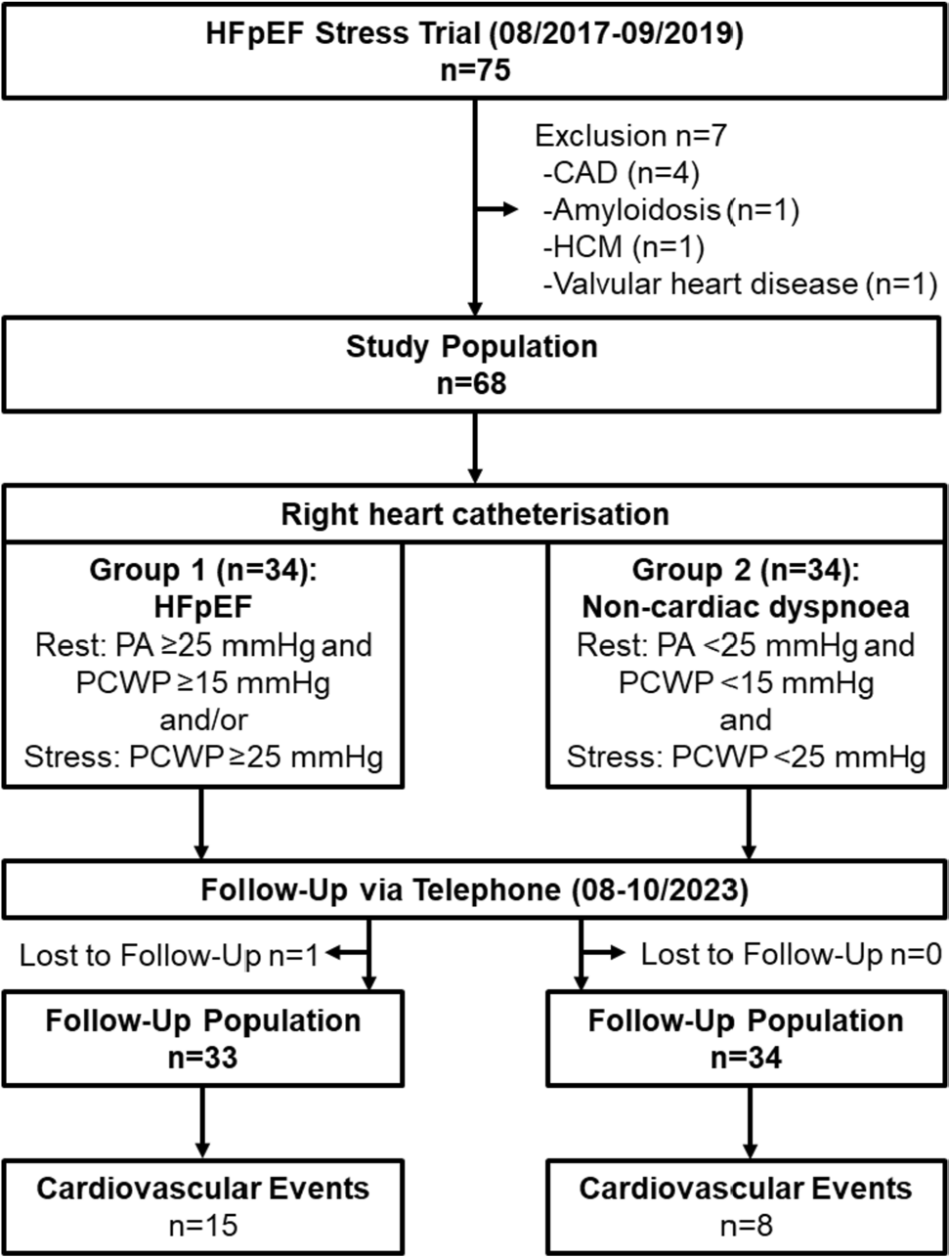
Study Flow-Chart. *HFpEF* heart failure with preserved ejection fraction, *CAD* coronary artery disease, *HCM* hypertrophic cardiomyopathy, *PA* pulmonary artery pressure, *PCWP* pulmonary capillary wedge pressure

The study population of the HFpEF Stress Trial (NCT03260621) was followed up via telephone interviews and medical records 4 years after baseline recruitment [12]. The clinical endpoint was CVE including cardiovascular hospitalisation and mortality. 75 patients with exertional dyspnea (NYHA class ≥ II) and signs of diastolic dysfunction (*E*/*e*’ ≥ 8) and preserved left ventricular ejection fraction (LVEF) ≥ 50% on echocardiography were prospectively recruited between 08/2017 and 09/2019. Exclusion criteria comprised contraindications for CMR as well as other causes of dyspnea including pulmonary (forced expiratory volume in 1 s or vital capacity < 80% of the reference) or cardiac (coronary artery disease as defined by a luminal stenosis ≥ 50%, ≥ moderate valvular heart disease) conditions. Because of new diagnoses other than HFpEF on CMR imaging, 7 patients were excluded from final analysis, Fig. 1. All patients underwent RHC, echocardiography and CMR at rest and during exercise-stress using supine bicycle ergometry as previously reported [12]. Data acquisition was performed 3 min after surpassing a heart rate of 100 beats/min at 50–60 rpm using a 5 Watt increasing ramp protocol.

RHC assessments included right atrial and ventricular (RA/RV) as well as pulmonary artery (PA) and capillary wedge pressures (PCWP). The presence of HFpEF was defined according to PCWP of ≥ 15 mmHg at rest or ≥ 25 mmHg during exercise-stress on RHC assessments. Furthermore, HFpEF patients were classified as masked HFpEF if diagnosed during exercise-stress only or overt HFpEF if diagnosed at rest. Patients were classified as NCD if PCWP did not meet the criteria for HFpEF and the absence of other cardiovascular diseases on CMR, echocardiography and RHC. Echocardiography was performed for apical 2, 3, and 4 chamber views (CV) as well as short axis (SA) views. Doppler analysis was performed for assessments of aortic, mitral and tricuspid valve regurgitation (colour), aortic outflow and tricuspid regurgitation velocities (continuous wave) and E/e’ in septal and lateral position (pulsed wave and tissue). Speckle-tracking echocardiography (STE) was performed on 2 and 4 CV orientations. The study was approved by the local ethics committee at the University Medical Center Goettingen. All patients gave written informed consent before participation. The study was conducted according to the principles of the Helsinki Declaration and funded by the German Centre for Cardiovascular Research (DZHK-17) [15].

### Cardiovascular magnetic resonance imaging

**Fig. 2:**
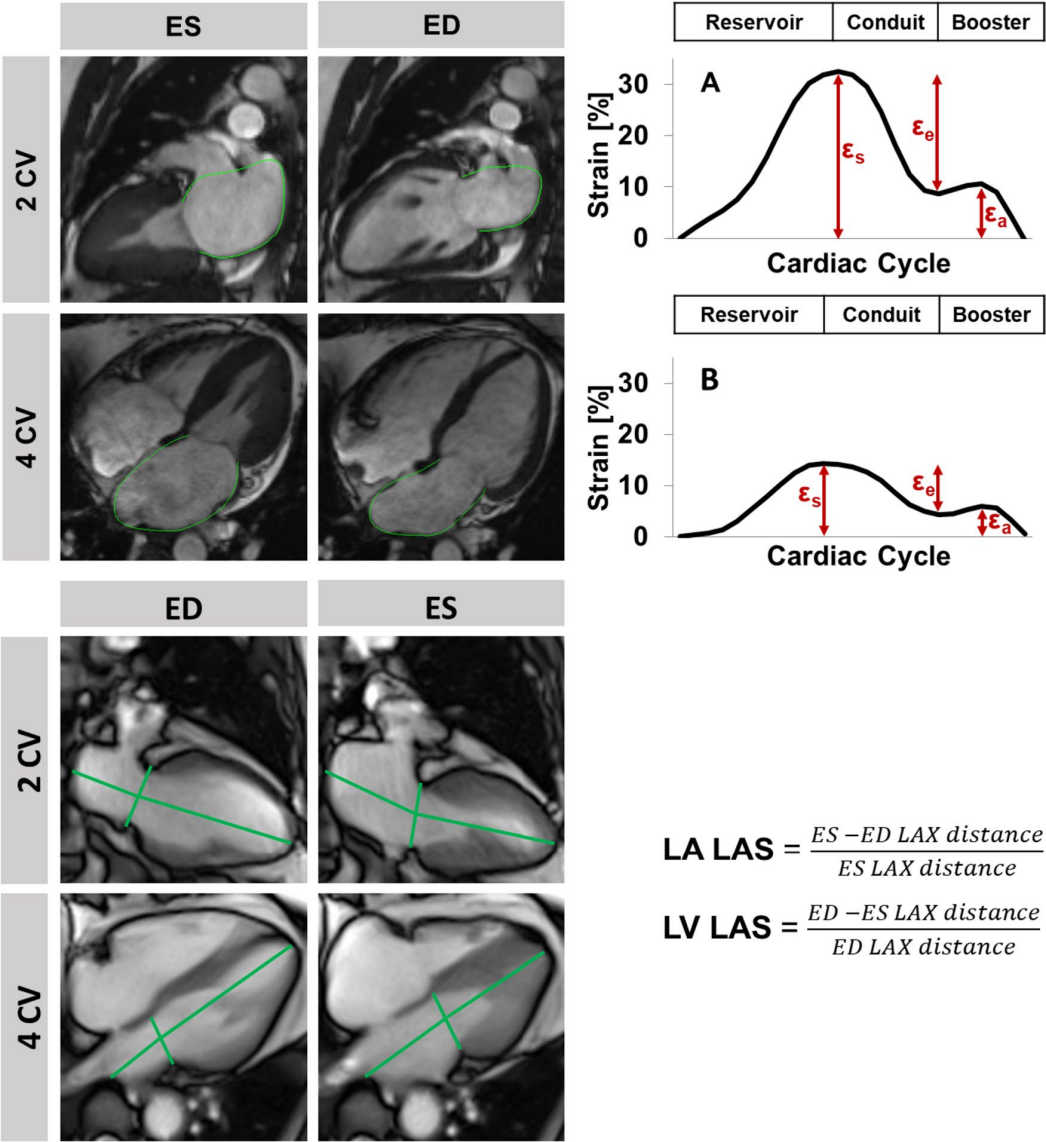
Feature-tracking and strain analysis. Top: On the left, left atrial (LA) end-systolic (ES) and -diastolic (ED) 2 and 4 chamber views (CV) with endocardial border tracking using cardiovascular magnetic resonance feature-tracking (CMR-FT). On the right, the corresponding strain curve of left atrial reservoir (εs), conduit (εe) and booster pump (εa) function for **A** a HFpEF patient without and **B** with cardiovascular event during follow-up. Bottom: Left atrial and ventricular (LV) long axis strain (LAS) assessment on a real-time CMR sequences shown at timespoints of ES and ED

At the time of cardiac imaging, all patients were in stable sinus rhythm. Myocardial function was assessed with conventional ECG triggered cine imaging at rest and free breathing RT-CMR imaging at rest and during physiological exercise. Conventional imaging at rest was performed using balanced steady state free precession (bSSFP) sequences including 2-, 3- and 4- chamber views (CV) as well as a short axis (SA) stack covering the entire heart. Post processing included volumetric analyses and tissue characterisation using Medis (QMass^®^, Medical Imaging Systems, Leiden, Netherlands) as well as feature-tracking (FT) deformation imaging using TomTec (2D CPA MR, Cardiac Performance Analysis, TomTec Imaging Systems, Unterschleissheim, Germany) [16]. Global longitudinal strain (GLS) was assessed in long axis views, global circumferential and radial strains (GCS/GRS) were assessed in SA stacks, respectively. Atrial phasic function was analysed in 2 and 4 CV and classified according to reservoir (total strain Es), passive conduit (Ee) and active booster pump (Ea) function [17, 18], Fig. 2. RT-CMR imaging was performed at rest and during exercise-stress using a bSSFP sequence combined with an undersampled radial encoding scheme [13]. Left atrial (LA) and ventricular (LV) long axis strains (LAS) were assessed in the 2 and 4 CV using OsiriX MD (Pixmeo SARL, CH-1233 Bernex, Switzerland) [19, 20].

Tissue characterisation was based on Modified Look-Locker Inversion recovery (MOLLI) sequences (pre- (5(3)3) and post-contrast (4(1)3(1)2) application) including septal and myocardial T1 times in one midventricular SA slice with subsequent calculation of extracellular volume (ECV) [21]. Late gadolinium enhancement (LGE) imaging was performed using inversion-recovery gradient echo sequences. Post-contrast images were obtained 10–20 min after the administration of gadolinium-based contrast agent (0.15 mmol/kg).

### Statistical analyses

Categorical variables are reported as frequencies and corresponding percentages and were compared using the chi-squared test. Continuous variables were tested for normal distribution using the Shapiro–Wilk test, are reported as median with associated interquartile ranges (IQR) and were compared using the nonparametric Mann–Whitney *U* test. Patients characteristics are reported according to the occurrence of CVE. Predictors for the latter were identified from Cox regression analyses, the results of which are reported as hazard ratios (HR) with 95% confidence intervals (CI), as well as areas under the ROC curve (AUC) analyses reported with 95% CI. AUCs were compared using the method proposed by DeLong et al. [22]. A 2-tailed *p* value < 0.05 was considered statistically significant. Analyses were performed using SPSS version 26.0 (IBM, Armonk, New York, USA) and MedCalc version 18.2.1 (MedCalc Software bvba, Ostend, Belgium).

## Results

### Study population

The study population consisted of 68 patients (HFpEF *n = *34, non-cardiac dyspnea (NCD) *n = *34) from the HFpEF Stress Trial as shown in Fig. 1. One patient was lost to follow-up. Fifteen patients with HFpEF (heart failure *n = *5, arrhythmia *n = *8, hypertension *n = *2) and eight with non-cardiac dyspnea (CAD including AMI, PCI and catheterisation *n = *4, arrhythmia *n = *3, heart failure *n = *1) were hospitalised due to cardiovascular reasons (*p = *0.059). Two patients had died, one of which due to cardiovascular reasons (heart failure hospitalisation followed by death). Cardiovascular hospitalisation and mortality are both considered in CVE. There were significantly more CVE in overt HFpEF (*n = *8/15) compared to NCD (*n = *8/26, *p = *0.040). Baseline characteristics according to CVE are reported in Table 1. Hospitalised patients were slightly older (71 vs. 67, *p = *0.030), while sex and cardiovascular risks factors were similar compared to patients without CVE during follow-up (*p ≥ *0.345). Patients with CVE during follow-up had a higher H2FPEF [23] (heavy, 2 or more antihypertensive drugs, atrial fibrillation, pulmonary hypertension, elder age > 60, elevated filling pressures; 5 vs. 3, *p < *0.001) but not HFA-PEFF (Heart Failure Association pretest assessment, echocardiography and natriuretic peptide, functional testing, final aetiology; 5 vs. 4, *p = *0.103) scores.

**Table 1:** Patients characteristics

Variable	Study population *n = *67*	Cardiovascular events *n = *23*	No cardiovascular events *n = *44*	*p* value
Age (years)	69 (64, 73)	71 (67, 75)	67 (58, 73)	**0.030**
Sex male/female	24/43	10/13	14/30	0.345
NYHA class	47 × II (70%) 20 × III (30%)	15 × II (65%) 8 × III (35%)	32 × II (73%) 12 × III (27%)	0.524
Atrial Fibrillation	21 (31%)	14 (61%)	7 (16%)	** < 0.001**
H2FPEF Score	4 (3, 5)	5 (4, 7)	3 (2, 5)	** < 0.001**
HFA-PEFF Score	4 (3, 6)	5 (3, 6)	4 (2, 5)	0.103
Cardiovascular risk factors				
Active smoking	9 (13%)	4 (17%)	5 (11%)	0.492
Hypertension	53 (79%)	18 (78%)	35 (80%)	0.902
Hyperlipoproteinemia	42 (63%)	15 (65%)	27 (61%)	0.757
Diabetes	9 (13%)	4 (17%)	5 (11%)	0.492
Body mass index (kg/m^2^ BSA)	28.1 (26.1, 32.7)	28.9 (26.8, 32.7)	27.7 (25.4, 32.8)	0.492
Laboratory testing				
NT-proBNP (ng/l)	123 (68, 268)	191 (108, 430)	88 (62, 180)	**0.005**
Creatinine (mg/dl)	0.85 (0.73, 1.03)	1.03 (0.83, 1.12)	0.78 (0.71, 0.98)	**0.002**
Echocardiography				
*E*/*e*ʹ rest	10.7 (9.0, 12.8)	11.4 (9.4, 13.0)	10.0 (8.5, 12.8)	0.132
*E*/*e*' stress	12.1 (10.3, 15.3)	12.1 (10.7, 14.5)	12.2 (10.0, 15.6)	0.941
LAVI (ml/m^2^ BSA)	38.2 (34.1, 50.0)	46.0 (38.4, 59.6)	36.1 (30.6, 43.3)	** < 0.001**
TAPSE (mm)	23.3 (20.8, 26.5)	21.7 (19.4, 27.2)	23.7 (21.7, 26.4)	0.349
PAPsys (mmHg)	24.2 (21.5, 30.3)	27.2 (21.7, 31.0)	23.9 (21.0, 29.2)	0.194
Right heart catheterization				
PCWP rest (mmHg)	11 (8, 14)	12 (9, 18)	10 (6, 13)	**0.018**
PCWP stress (mmHg)	23 (18, 27)	26 (21, 30)	22 (14, 26)	**0.020**
PA mean rest (mmHg)	19 (16, 23)	21 (19, 26)	18 (15, 21)	**0.005**
PA mean stress (mmHg)	39 (33, 44)	43 (38, 50)	37 (30, 42)	**0.003**
PA pO_2_ rest (%)	74 (71, 77)	72 (70, 76)	75 (72, 77)	0.095
PA pO_2_ stress (%)	47 (39, 51)	46 (35, 50)	47 (42, 52)	0.282
Cardiac Index rest (l/m^2^ BSA)	2.9 (2.5, 3.2)	2.7 (2.5, 3.6)	2.9 (2.6, 3.2)	0.602
Cardiac Index stress (l/m^2^ BSA)	5.3 (4.3, 6.4)	5.6 (4.0, 6.3)	5.3 (4.3, 6.4)	0.787

NYHA: New York Heart Association, LAVI: left atrial volume index, TAPSE: tricuspid annular plane systolic excursion, PAPsys: systolic pulmonary artery pressure, PCWP: pulmonary capillary wedge pressure, PA: pulmonary artery pressure, BSA: body surface area. Comparisons were made between patients with and without cardiovascular event. Categorical parameters are reported in absolutes numbers and were compared using the Chi-squared test. Independent continuous parameters are presented as medians with interquartile ranges and were compared using the Mann–Whitney *U* test. Bold *p* values indicate statistical significance. Baseline characteristics have been published previously [12]

^a^Numbers differ for echocardiographic assessments shown for study population/with/without hospitalisation (*E*/*e*ʹ stress *n = *49/17/32; TAPSE *n = *59/20/39;PAPsys *n = *56/19/37)

On echocardiography patients with CVE showed no difference for E/e’ in echocardiography at rest (*p = *0.132), the left atrial volume index (LAVI) was significantly increased (*p = * < 0.001) and AF was significantly more frequent in these patients (14/23, 61% with vs. 17/44, 16% without CVE, *p < *0.001).

On RHC patients with CVE had higher PCWP and mean pulmonary artery (PA) pressures at rest and during exercise-stress (*p ≤ *0.020).

### Functional alterations

STE, FT- and RT-CMR deformation assessment parameters are reported in Table 2 according to CVE. LV function assessed by STE CMR-FT deformation imaging and RT-CMR LAS revealed no differences comparing patients with and without CVE during follow-up (*p ≥ *0.146) with the exception of LV LAS at rest only, being impaired in patients with CVE (*p = *0.028).

**Table 2:** Non-invasive rest and exercise-stress imaging

Variable	Cardiovascular events	No cardiovascular events	*p* value
	*n = *23^a^	*n = *44^a^	
Echocardiography			
STE LV GLS rest	− 15.9 (− 11.9, − 18.9)	− 15.5 (− 13.2, − 19.2)	0.899
STE LV GLS stress	− 14.4 (− 9.3, − 17.0)	− 15.5 (− 13.8, − 19.7)	0.233
STE LA Es rest	15.9 (10.2, 25.5)	30.3 (25.9, 33.6)	** < 0.001**
STE LA Es stress	15.3 (9.6, 27.3)	29.0 (21.4, 36.4)	**0.004**
Conventional cardiovascular magnetic resonance			
FT LV GLS	− 19.8 (− 18.7, − 22.3)	− 20.8 (− 19.2, − 23.3)	0.205
FT LV GCS	− 36.4 (− 29.4, − 39.4)	− 34.4 (− 30.9, − 37.1)	0.501
FT LV GRS	68.3 (60.1, 74.6)	62.8 (52.6, 70.9)	0.066
FT RV GLS	− 24.6 (− 20.4, − 26.6)	− 22.8 (− 19.9, − 26.1)	0.376
FT LA Es	23.1 (14.3, 30.8)	33.9 (27.9, 40.6)	** < 0.001**
FT LA Ee	9.9 (7.8, 13.2)	16.4 (12.0, 21.6)	** < 0.001**
FT LA Ea	11.3 (6.6, 16.4)	16.9 (13.4, 20.9)	**0.002**
Native T1 myocardium (ms)	1196 (1183, 1224)	1212 (1186, 1248)	0.224
Native T1 septum (ms)	1208 (1167, 1231)	1203 (1179, 1230)	0.792
ECV myocardium	25.9 (24.0, 28.2)	25.6 (24.2, 27.5)	0.840
ECV septum	25.0 (23.2, 28.4)	25.3 (23.3, 26.9)	0.962
Real-time cardiovascular magnetic resonance			
LV LAS Rest	13.2 (11.2, 14.2)	14.2 (12.1, 16.2)	**0.028**
LV LAS Stress	16.1 (12.6, 19.0)	18.0 (14.4, 20.0)	0.146
LA EF Rest	32.0 (22.3, 37.8)	38.8 (34.3, 43.1)	**0.002**
LA EF Stress	32.3 (19.0, 42.7)	42.2 (35.9, 49.5)	**0.002**
LA LAS Rest	13.7 (9.3, 17.8)	21.6 (17.2, 26.3)	** < 0.001**
LA LAS Stress	17.6 (9.0, 26.3)	25.7 (18.4, 30.2)	**0.006**

All values are reported in % unless stated otherwise. Independent continuous parameters are presented as medians with interquartile ranges and were compared using the Mann–Whitney U test. Bold *p* values indicate statistical significance

*STE* speckle tracking echocardiography, *LV* left ventricular, *LA* left atrium, *FT* feature-tracking, *GLS/GCS/GRS* global longitudinal/circumferential/radial strain, *Es/Ee/Ea* atrial reservoir/conduit/booster pump function, *LAS* long axis strain, *EF* ejection fraction

^a^Numbers differ for echocardiographic assessments shown for study with/without hospitalisation (LV GLS rest *n = *17/35, LV GLS stress *n = *15/30, LA Es *n = *17/36 and LA Es stress *n = *15/31)

In contrast, LA function was impaired in patients with CVE. This included STE at rest (*p < *0.001) and stress (*p = *0.004), phasic function using CMR-FT (Es *p < *0.001, Ee *p < *0.001, Ea *p = *0.002) as well as rest and exercise-stress RT-CMR derived LA EF (rest/stress: *p = *0.002) and LA LAS (rest: *p < *0.001, exercise-stress *p = *0.006).

### Prognostic implication

Hazard ratios for CVE and accuracies to predict CVE are reported in Table 3. An increase in the H2FPEF (HR 1.49, *p = *0.006) but not in the HFA-PEFF (HR 1.29, *p = *0.075) score was significantly associated with higher CVE rates. Visually, there was no LGE present. Tissue characterisation showed no association to outcome. STE, CMR-FT and RT-CMR LAS showed no significant association between LV function parameters and risk of CVE (*p ≥ *0.171, AUC ≤ 0.64) except for LV LAS at rest (HR 0.84, *p = *0.029).

**Table 3:** Prognostic estimation and diagnostic accuracy

Variable	Hazard ratio (95% CI)	*p* value	AUC^a^
Clinical			
Age	1.05 (1.00–1.11)	0.041	0.66 (0.53–0.79)
H2FPEF Score	1.49 (1.23–1.81)	** < 0.001**	0.79 (0.69–0.90)
HFA-PEFF	1.29 (0.97–1.71)	0.075	0.62 (0.48–0.76)
Laboratory testing			
NT-proBNP (ng/l)	1.00 (1.00–1.00)	** < 0.001**	0.71 (0.58–0.84)
Echocardiography^a^			
*E*/*e*ʹ rest	1.07 (1.01–1.15)	**0.035**	0.61 (0.47–0.76)
LAVI (ml/m^2^ BSA)	1.05 (1.02–1.07)	** < 0.001**	0.77 (0.65–0.88)
STE LV GLS rest	1.03 (0.91–1.16)	0.697	0.51 (0.34–0.69)
STE LV GLS exercise	1.02 (0.95–1.09)	0.636	0.61 (0.44–0.78)
STE LA Es rest	0.88 (0.84–0.93)	** < 0.001**	0.86 (0.73–0.98)
STE LA Es exercise	0.92 (0.87–0.97)	**0.004**	0.77 (0.62–0.91)
Right heart catheterisation			
PCWP rest (mmHg)	1.13 (1.04–1.23)	**0.004**	0.68 (0.55–0.81)
PCWP stress (mmHg)	1.06 (1.01–1.10)	**0.013**	0.67 (0.54–0.81)
PA mean rest (mmHg)	1.08 (1.02–1.14)	**0.005**	0.71 (0.58–0.84)
PA mean stress (mmHg)	1.04 (1.01–1.08)	**0.006**	0.72 (0.59–0.85)
Conventional cardiovascular magnetic resonance			
FT LV GLS	1.08 (0.96–1.22)	0.203	0.60 (0.44–0.75)
FT LV GCS	1.00 (0.98–1.02)	0.822	0.55 (0.40–0.70)
FT LV GRS	1.02 (0.99–1.04)	0.171	0.64 (0.50–0.77)
FT RV GLS	0.96 (0.89–1.03)	0.253	0.57 (0.42–0.72)
FT LA Es	0.92 (0.89–0.96)	** < 0.001**	0.78 (0.65–0.91)
FT LA Ee	0.87 (0.80–0.94)	** < 0.001**	0.76 (0.64–0.89)
FT LA Ea	0.91 (0.85–0.97)	**0.003**	0.73 (0.59–0.87)
Real-time cardiovascular magnetic resonance			
LV LAS Rest	0.84 (0.72–0.98)	**0.029**	0.66 (0.53–0.80)
LV LAS Stress	0.92 (0.83–1.02)	0.323	0.61 (0.46–0.76)
LA EF Rest	0.93 (0.89–0.96)	** < 0.001**	0.74 (0.60–0.87)
LA EF Stress	0.93 (0.90–0.97)	** < 0.001**	0.73 (0.60–0.86)
LA LAS Rest	0.86 (0.80–0.93)	** < 0.001**	0.80 (0.67–0.93)
LA LAS Stress	0.92 (0.88–0.97)	**0.002**	0.71 (0.56–0.85)

All values are reported in % unless stated otherwise. Hazard rations (HR) for the occurrence of cardiovascular events were calculated using Cox regression analyses

*NT-proBNP* N-terminal prohormone of brain natriuretic peptide, *LAVI* left atrial volume index, *STE* speckle tracking echocardiography, *LV* left ventricular, *LA* left atrium, *PCWP* pulmonary capillary wedge pressure, *PA* pulmonary artery pressure, *FT* feature-tracking, *GLS/GCS/GRS* global longitudinal/circumferential/radial strain, *Es/Ee/Ea* atrial reservoir/conduit/booster pump function, *LAS* long axis strain, *EF* ejection fraction

^a^Numbers differ for echocardiographic assessments (LV GLS rest *n = *52, LV GLS stress *n = *45, LA Es *n = *53 and LA Es stress *n = *46

**Fig. 3:**
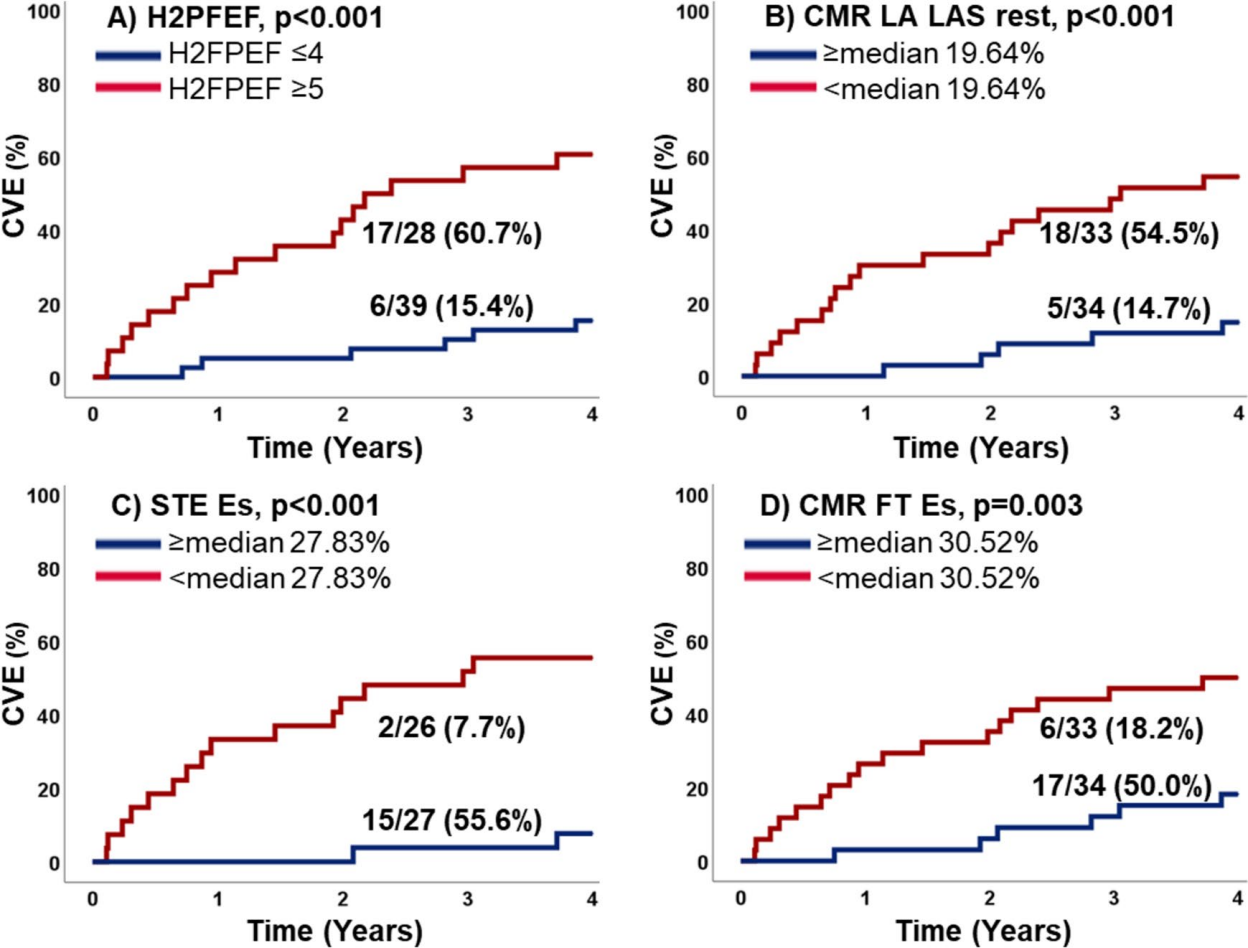
Cardiovascular events during follow-up. The graph shows the percentage of patients with cardiovascular events (CVE) in patients with **A** H2FPEF score ≤ 4 and ≥ 5 points, **B** cardiovascular magnetic resonance imaging (CMR) derived left atrial long axis strain (LA LAS) at rest above or below the median, **C** speckle tracking echocardiography (STE) derived left atrial reservoir strain (Es) at rest above or below the median and **D** CMR-feature tracking (FT) derived left atrial reservoir strain (Es) at rest above or below the median

Impaired atrial function at rest was associated with increased likelihood for CVE in Cox-regression analyses using STE, CMR-FT and RT-CMR (*p ≤ *0.003) and during exercise-stress (*p ≤ *0.004). This can further be appreciated from Kaplan–Meier plots after dichotomisation at the median, Fig. 3. These results remained significant in the subgroup of patients with invasively proven HFpEF (*p ≤ *0.020). LA functional testing at rest using STE (*p = *0.008), CMR-FT LA Es/Ee (*p = *0.016/0.017) and RT-CMR derived LAS (*p = *0.003) were independent predictors of CVE regardless of the presence of AF.

**Fig. 4:**
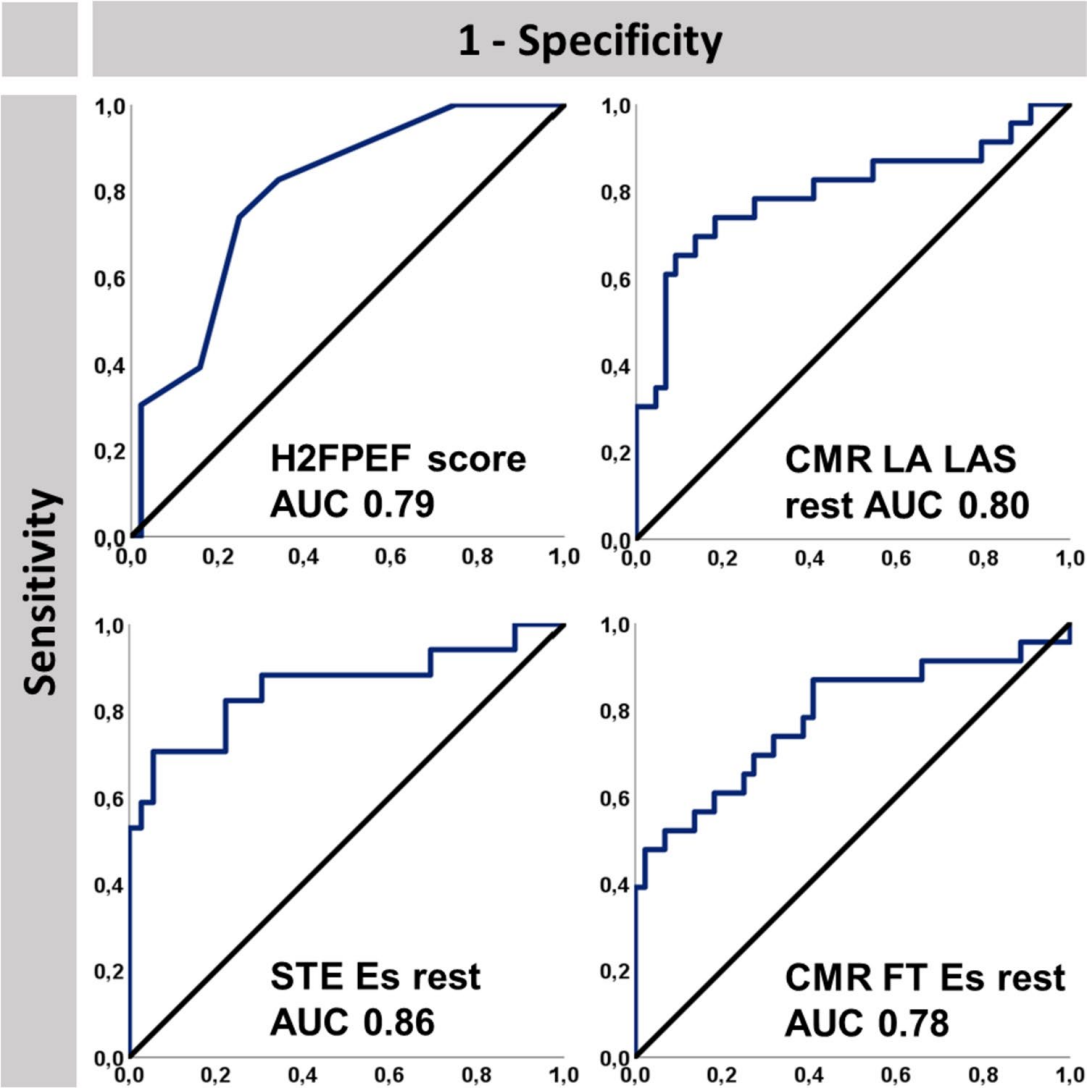
Accuracy to predict cardiovascular events. The figure displays the accuracy to predict cardiovascular events as areas under the ROC curve (AUC) for the H2FPEF score as well as cardiovascular magnetic resonance imaging (CMR) derived left atrial (LA) long axis strain (LAS), speckle tracking echocardiography (STE) derived reservoir strain (Es) and CMR-derived Es at rest

The highest accuracy for the prediction of CVE was found for LA deformation imaging (AUC STE Es 0.86, CMR-FT Es 0.78, and RT CMR LAS 0.80), Fig. 4.

Subgroup analyses for NCD, masked and overt HFpEF revealed that only STE Es (*p = *0.016/0.032) and LA LAS at rest (*p = *0.037/0.017) have prognostic implications both in masked and overt HFpEF, Table 4. Prognostic implications remained significant in overt but not masked HFpEF independent of atrial fibrillation; LA LAS (overt HFpEF: HR 0.80, 95% CI 0.67–0.96, *p = *0.018, masked HFpEF: HR 0.94, 95% CI 0.83–1.06, *p = *0.287) and STE Es (overt HFpEF: HR 0.87, 95% CI 0.76–1.00, *p = *0.042, masked HFpEF: HR 0.96, 95% CI 0.87–1.05, *p = *0.334).

**Table 4:** Prognostic implications of atrial functional assessments according to subgroups

Variable	NCD *n = *34^a^ HR (95% CI), *p* value	masked HFpEF *n = *18* HR (95% CI), *p* value	overt HFpEF *n = *15* HR (95% CI), *p* value
Echocardiography			
STE LA Es rest	0.80 (0.67–0.96), ***p = *0.018**	0.89 (0.81–0.98), ***p = *0.016**	0.88 (0.78–0.99), ***p = *0.032**
STE LA Es exercise	0.96 (0.87–1.06), *p = *0.422	0.91 (0.81–1.03), *p = *0.137	0.91 (0.82–1.02), *p = *0.120
Conventional CMR at rest			
FT LA Es	0.86 (0.74–0.99), ***p = *0.038**	0.95 (0.88–1.02), *p = *0.157	0.93 (0.86–1.01), *p = *0.074
FT LA Ee	0.85 (0.73–1.00), *p = *0.054	0.93 (0.81–1.07), *p = *0.326	0.87 (0.74–1.02), *p = *0.095
FT LA Ea	0.93 (0.81–1.08), *p = *0.335	0.94 (0.86–1.04), *p = *0.230	0.90 (0.77–1.05), *p = *0.165
RT-CMR at rest and stress			
LA EF Rest	0.90 (0.80–1.01), *p = *0.080	0.91 (0.83–1.00), *p = *0.050	0.97 (0.91–1.03), *p = *0.310
LA EF Stress	0.91 (0.82–1.01), *p = *0.071	0.91 (0.84–0.99), ***p = *0.034**	0.97 (0.91–1.04), *p = *0.434
LA LAS Rest	0.89 (0.78–1.02), *p = *0.101	0.88 (0.78–0.99), ***p = *0.037**	0.80 (0.67–0.96), ***p = *0.017**
LA LAS Stress	0.96 (0.84–1.09), *p = *0.501	0.82 (0.71–0.95), ***p = *0.009**	0.96 (0.86–1.07), *p = *0.454

Hazard ratios (HR) were calculated by the means of cox regression analyses. Bold p-values indicate statistical significance

*NCD* non-cardiac dyspnoea, *HFpEF* heart failure with preserved ejection fraction, *STE* speckle-tracking echocardiography, *LA* left atrium, *CMR* cardiovascular magnetic resonance, *FT* feature-tracking, *Es/Ee/Ea* atrial reservoir/conduit/booster pump function, *EF* ejection fraction, *LAS* long axis strain

^a^*n* numbers vary for echocardiographic assessments at rest/during exercise-stress: NCD: (*n = *25/20), masked HFpEF: (*n = *16/15), overt HFpEF: (*n = *12/11)

## Discussion

The HFpEF Stress Trial has demonstrated high accuracy of RT-CMR physiological exercise-stress testing for the diagnosis of HFpEF [12]. The current analyses include the clinical 4-year follow-up of this cohort and demonstrate an association of LA function with outcome. Atrial functional failure determined by LA LAS during exercise-stress unmasks post capillary pulmonary hypertension for optimized non-invasive identification of HFpEF patients. In addition, atrial functional failure both at rest and during exercise-stress is associated with worse prognosis. Notwithstanding, LA dysfunction at rest provides the best accuracy for the prediction of CVE. Both STE Es and RT-CMR LA LAS yield high prognostic accuracy and were the only parameters with predictive value in both subgroups of overt and masked HFpEF; however, high quality STE for post-processing was only obtained in 80% of patients at rest and 68% during exercise-stress. Based on the previously published HFpEF Stress trial [12] and the current clinical follow-up data, we recommend that comprehensive CMR HFpEF assessments should incorporate atrial function quantification at rest and exercise-stress for the diagnostic and prognostic evaluation of HFpEF.

### CMR for comprehensive assessment of cardiac pathology in HFpEF

CMR offers a comprehensive and non-invasive approach for the assessment of cardiac pathophysiology in HFpEF using tissue characterisation and function quantification [24–26]. The STIFFMAP Trial [27] demonstrated that CMR derived tissue characterisation independently predicts invasively assessed LV stiffness by pressure–volume loops. CMR deformation imaging allows the assessment of myocardial contractility and relaxation. CMR-FT derived LV GLS has been shown to correlate with invasively assessed LV relaxation Tau [28] and is associated with heart failure hospitalisation and mortality [29]. LA function has also been shown to be a sensitive imaging-biomarker for cardiac remodelling in HFpEF [30, 31] independent of atrial size [32] and associated with cardiovascular outcome [33]. CMR-FT atrial assessments offer differentiation of the three phases of atrial physiology, which can be attributed to atrial elasticity (reservoir and to some extent conduit function) and function parameters that contribute to LV filling (booster pump and to some extent conduit function) [17]. These phases have been shown to carry distinct physiological information with direct clinical and prognostic information. Atrial reservoir function, representing the collection of venous return during ventricular systole and elasticity of the atrium, is associated with cardiovascular mortality; for example, following acute myocardial infarction independent of ventricular function and tissue composition [34]. Passive atrial restoring forces and early diastolic ventricular filling are quantified by atrial passive conduit strain which has been shown to be associated with exercise intolerance in HFpEF [35] independent of LV stiffness and relaxation. LA active contractility (booster pump strain) has been reported to compensate for LV heart failure [36]. The finding that LA reservoir strain by both STE and CMR-FT provides high prognostic information regarding CVE may indicate that atrial elasticity by reducing pulmonary venous hypertension has stronger prognostic impact than LA function parameters contributing to LV filling (booster pump strain and to some extent conduit strain). We speculate of a prominent role of active atrial contractility during exercise as well as elasticity at rest in HFpEF pathophysiology. While exercise induced impaired active atrial contractility can precisely identify patients with HFpEF, impaired elasticity at rest identifies those patients at risk for hospitalisation due to congestion at rest or minimal levels of exercise at later disease stages. This is paralleled by data from Melenovsky et al. [37] who demonstrated that echocardiography derived active atrial contractility as defined by A’ from tissue velocity mapping during exercise was impaired and so a potential mechanism of cardio pulmonary congestion in HFpEF. Indeed, exercise-stress testing emerged as a cornerstone in the early diagnosis of HFpEF [6, 7]. Advances in CMR imaging introduced in the HFpEF Stress Trial [12, 13] demonstrated that RT-CMR imaging allows the transition of exercise-stress testing into the already broad spectrum of CMR imaging. Although RT-CMR does not allow for detailed deformation imaging assessment of the three atrial phases, our results show that it accurately reflects LA longitudinal function (and potentially increased active contractility during exercise), which allows for the identification of early stage HFpEF based on surrogate estimation (LV/LA LAS) of global LV and LA longitudinal strains [20]. This allows the translation of longitudinal deformation imaging to physiological exercise-stress assessments [12].

### Rest and exercise-stress assessment for diagnostic and prognostic stratification in HFpEF

Even in the presence of exertional dyspnea, euvolemic HFpEF patients may present with normal natriuretic peptides and cardiac filling pressures at rest [6]. Exercise-stress induces atrial failure and congestion unmasking pathology in early stages of cardiac remodelling [38], thus allowing for the diagnosis of HFpEF. The HFpEF Stress Trial demonstrated assessment of exercise-induced atrial failure using LA LAS as the most precise parameter for the non-invasive identification of invasively proven HFpEF [12]. In the present study, atrial phasic function at rest, specifically reservoir function, and LA LAS at rest were identified as powerful predictors of CVE. Indeed, patients with advanced stages of cardiac remodelling and impaired atrial function at rest were more likely to be hospitalised during medium-term follow-up of 4 years. Based upon the presented data, for prognostic implications only, e.g., in known HFpEF, a shortened protocol for atrial function quantification including either conventional breath-hold bSSFP, novel free-breathing real-time CMR or echocardiography-based deformation imaging could be employed.

Importantly, in the present population, atrial function assessment (STE Es, CMR-FT Es/Ee and RT-CMR LA LAS) was associated with CVE independently of known AF in the medical history. Indeed, increased preload in diastolic dysfunction leads to atrial remodelling, dilatation and fibrillation [39]. AF burden further promotes atrial remodelling inducing progressive deterioration of atrial mechanics with subsequent worsening of cardiac haemodynamics [40, 41]. The distinct role of AF is also reflected in the H2FPEF score, with AF being the only factor accounting for 3 points [23]. Notwithstanding, atrial dysfunction is not limited to AF, the term atrial cardiomyopathy [42] has recently been introduced to describe intrinsic atrial dysfunction in cardiovascular diseases.

In our cohort at an early disease stage, 56% of HFpEF patients were identified by PCWP exercise-stress thresholds only [12]. Left atrial reservoir and conduit functions had higher diagnostic accuracy for CVE compared to booster (contractility) function. Indeed, reports in HFpEF indicate that changes in conduit function precede reduced atrial contractility, which initially shows an increase in active contractility in the early stages of diastolic dysfunction, compensating for increased LV filling pressures [43]. At later stages the contractility decreases as the disease progresses [44]. Notwithstanding, the assessment of a surrogate for global atrial function (LA LAS) on one hand side reliably detects an exercise induced atrial failure allowing the diagnosis of HFpEF and on the other hand side allows accurate risk stratification in the 4-year follow-up of our trial. The 2-year follow-up of the present population [14] had shown that a more in-depth assessment of the relative phasic function at rest provided the highest diagnostic accuracy. Indeed, as HFpEF tends to have slower progression of symptoms and disease severity compared to HFrEF, impaired phasic function may reflect atrial functional failure more accurately and thus may have the better short-term prognostic accuracy as these patients may suffer from CVE in the nearer future. For a long-term prognosis, overall atrial longitudinal function provides high diagnostic and prognostic accuracy. Importantly, to date, new therapeutic approach for HFpEF has demonstrated significant morbidity reduction [4], while it is important to note that delayed diagnosis may negate prognostic benefits [5, 45]. Atrial functional failure represents a composite of innate atrial functional loss [42] and the burden of LV congestion with increased filling pressures [46], the latter being closely related to symptom onset in HFpEF [47] highlighting its role in HFpEF.

In the current trial, an even longer period of follow-up (or higher patient numbers) may have led to a larger number of CVEs, and possibly a higher value for LV analyses for long-term prognosis prediction as previously demonstrated by Park et al. [29]. LV LAS at rest was the only ventricular parameter associated with CVE. This may again be due to the fact that patients included were at an early disease stage. This also highlights that progress in cardiac remodelling, once apparent in the LV, would significantly increase the risk of CVE. Future research, including a larger cohort of HFpEF patients further down the line of cardiac remodelling with a longer follow-up period, is required to fully understand the clinical significance and the prognostic impact of CMR-derived left atrial and ventricular function at rest and during exercise-stress. For the time being, the atrium reflects the ideal chamber for early diagnostic and prognostic assessments.

## Study limitations

The HFpEF Stress Trial investigated a newly developed diagnostic test in an experienced CMR core-laboratory. Conclusions derived from this follow-up study, therefore, represents single center experience with a relatively small study population. Fifty-six percent of HFpEF patients were identified by exercise-stress testing only. As this may represent relatively early disease stages, a follow-up of 4 years may still be to short to allow for the development of adverse remodelling and the occurrence of CVE. Notwithstanding we were able to identify LA functional impairment at rest to be associated with the highest accuracy for CVE prediction in this population. Patient classification was based on PCWP and PA pressures only [7], while recently described invasively assessed criteria were not considered at the time of recruitment [48].

## Conclusion

This follow-up study of the HFpEF Stress Trial demonstrates that left atrial function both at rest and during exercise-stress is associated with worse prognosis in HFpEF. However, atrial failure at rest is associated with disease progression and yields the highest prognostic value for the prediction of CVE. LA LAS allows easy and software independent approximation of LA longitudinal strain. Consequently, RT-CMR rest and exercise-stress LAS quantification enables both early diagnosis as well as prognostic estimation in HFpEF patients. A multi-center approach is warranted for further validation.

## Data Availability

The data underlying the findings is available at the imaging database of the German Centre for Cardiovascular Research (DZHK) and access will be granted to researchers that meet the criteria for access upon formal request.

## Abbreviations

AF: Atrial fibrillation
CMR: Cardiovascular magnetic resonance
EF: Ejection fraction
FT: Feature tracking
GLS: Global longitudinal strain
HFpEF: Heart failure with preserved ejection fraction
LA: Left atrium
LAS: Long axis strain
LV: Left ventricle
PCWP: Pulmonary capillary wedge pressure
RHC: Right heart catheterization
RT: Real time
STE: Speckle-tracking echocardiography
